# Regulation of Interleukin-6 Receptor Signaling by TNF Receptor-Associated Factor 2 and 5 During Differentiation of Inflammatory CD4^+^ T Cells

**DOI:** 10.3389/fimmu.2018.01986

**Published:** 2018-08-30

**Authors:** Hiroyuki Nagashima, Naoto Ishii, Takanori So

**Affiliations:** ^1^Lymphocyte Cell Biology Section, Molecular Immunology and Inflammation Branch, National Institute of Arthritis, Musculoskeletal and Skin Diseases, National Institutes of Health, Bethesda, MD, United States; ^2^Department of Microbiology and Immunology, Tohoku University Graduate School of Medicine, Sendai, Japan; ^3^Laboratory of Molecular Cell Biology, Graduate School of Medicine and Pharmaceutical Sciences, University of Toyama, Toyama, Japan

**Keywords:** TRAF5, TRAF2, IL-6, T_H_17, autoimmunity, inflammation

## Abstract

There is growing evidence that tumor necrosis factor (TNF) receptor-associated factors (TRAFs) bind to unconventional membrane-bound receptors in many cell types and control their key signaling activity, in both positive and negative ways. TRAFs function in a variety of biological processes in health and disease, and dysregulation of TRAF expression or activity often leads to a patho-physiological outcome. We have identified a novel attribute of TRAF2 and TRAF5 in interleukin-6 (IL-6) receptor signaling in CD4^+^ T cells. TRAF2 and TRAF5 are highly expressed by naïve CD4^+^ T cells and constitutively bind to the signal-transducing receptor common chain gp130 via the C-terminal TRAF domain. The binding between TRAF and gp130 limits the early signaling activity of the IL-6 receptor complex by preventing proximal interaction of Janus kinases (JAKs) associated with gp130. In this reason, TRAF2 and TRAF5 in naïve CD4^+^ T cells negatively regulate IL-6-mediated activation of signal transducer and activator of transcription 3 (STAT3) that is required for the development of IL-17-secreting CD4^+^ T_H_17 cells. Indeed, *Traf2*-knockdown in differentiating *Traf5*^−/−^ CD4^+^ T cells strongly promotes T_H_17 development. *Traf5*^−/−^ donor CD4^+^ T cells exacerbate the development of neuroinflammation in experimental autoimmune encephalomyelitis (EAE) in wild-type recipient mice. In this review, we summarize the current understanding of the role for TRAF2 and TRAF5 in the regulation of IL-6-driven differentiation of pro-inflammatory CD4^+^ T cells, especially focusing on the molecular mechanism by which TRAF2 and TRAF5 inhibit the JAK-STAT pathway that is initiated in the IL-6 receptor signaling complex.

## Introduction

The tumor necrosis factor receptor-associated factor (TRAF) family molecules in mammals were initially discovered as cytoplasmic adaptor proteins interacting with one of the tumor necrosis factor receptor superfamily (TNFRSF) molecules, TNFR2 (1). TRAF molecules are also present in *Caenorhabditis elegans* and *Drosophila melanogaster* (2–4). There are six mammalian TRAF molecules, TRAF1 to TRAF6, which share a conserved C-terminal TRAF-C domain that accommodates a short stretch of amino acids found in the cytoplasmic tail of receptors. Mammalian TRAFs critically participate in the signal transduction by receptors, such as TNFRSF molecules, Toll-like receptors (TLRs), nucleotide binding-oligomerization domain (NOD)-like receptors (NLRs), retinoic acid-inducible gene (RIG)-I-like receptors (RLRs), interleukin receptors, interferon receptors, transforming growth factor-β (TGF-β) receptor, the T-cell receptor (TCR) and platelet receptors. TRAFs link these receptors to various signaling cascades that are important in health and disease (3, 5–12).

One of the TRAF family molecules, TRAF5, is highly expressed in lung and moderately expressed in thymus, spleen, and kidney (13). In contrast to mice deficient in *Traf2, Traf3*, or *Traf6*, which become runted and die prematurely, *Traf5*^−/−^ mice are born at the expected Mendelian ratios and exhibit no obvious abnormalities (14). One important question to be resolved is how TRAF5 specifically regulates cellular responses that are different and separate from those regulated by other TRAF family molecules.

Upon antigen exposure, naïve CD4^+^ T cells differentiate into different effector CD4^+^ helper T cell (T_H_ cell) subsets that control the functions of B cells, macrophages, and CD8^+^ cytotoxic T cells through cell-to-cell contact and/or by secreting specific effector cytokines. There is growing evidence that TRAFs recruited to the TCR, costimulatory TNFRSF molecules, and cytokine receptors control key signaling events in CD4^+^ T cells and are critical for the activation, differentiation, and survival of T_H_ cells in both positive and negative manners (11).

Although it has been well recognized that TRAF molecules play essential roles in T cell biology, the detailed functions and their molecular mechanisms of action are still enigmatic. In this review, we will highlight a novel function of TRAF2 and TRAF5 in the regulation of CD4^+^ T_H_17 cell differentiation that is controlled by pro-inflammatory cytokine IL-6 and its receptor signaling complex.

## TRAF2 and TRAF5 in IL-6 receptor signaling and T_H_17 development

The regulation of IL-6 receptor signaling by TRAF molecules was initially suggested by the observation that after culturing in IL-6-containing T_H_17 skewing condition *in vitro*, differentiating CD4^+^ T cells lacking *Traf5* produced a higher amount of IL-17 than did wild-type counterparts. However, *Traf5*-deficiency had no significant role for the development of T_H_1, T_H_2, T_H_17, Treg cells in polarized *in vitro* cultures. Accordingly, *Traf5*^−/−^ mice exhibited exacerbated T_H_17 cell-dependent neuroinflammation in a model of experimental autoimmune encephalomyelitis (EAE). The enhanced EAE phenotype was recapitulated in irradiated wild-type mice that had been transferred with *Traf5*^−/−^ CD4^+^ T cells, demonstrating that TRAF5 expressed in CD4^+^ T cells negatively regulates the generation of pathogenic T_H_17 cells (15). These results strongly suggested that TRAF5 regulated IL-6 receptor signaling that is required for T_H_17 differentiation.

Indeed, *Traf5*^−/−^ naïve CD4^+^ T cells stimulated with a complex of IL-6 and soluble IL-6R (IL-6–sIL-6R) without triggering of TCR and CD28 exhibited increased phosphorylation of JAK1 and STAT3. In addition, the retrovirally transduced *Traf5* gene in CD4^+^ T cells suppressed the phosphorylation of STAT3 mediated by IL-6–sIL-6R (16, 17). The negative regulatory function of TRAF5 for STAT3 was also observed in primary CD8^+^ T cells, but not in macrophages. One of the possible reasons would be that the expression of *Traf5* mRNA was almost five times lower in macrophages than in CD4^+^ and CD8^+^ T cells (15). These results strongly suggest that if a cell expresses substantial levels of endogenous TRAF5 and gp130, TRAF5 can repress IL-6 receptor signaling activity in this cell type. Importantly, TRAF5 exhibited no inhibitory role for the STAT3 phosphorylation mediated by signaling through IL-10 receptor or IL-21 receptor in CD4^+^ T cells, demonstrating the specific action of TRAF5 for IL-6 receptor signaling (15).

By using a BAF/B03 cell line that stably expresses gp130 (BAF-gp130), we examined the role for TRAF family molecules in IL-6 receptor signaling and found that not only TRAF5 but also TRAF2 inhibited STAT3 phosphorylation and cell proliferation mediated by IL-6–sIL-6R, while TRAF1, TRAF3, TRAF4, and TRAF6 did not. In accordance with this, TRAF2 displayed a similar activity as TRAF5 in terms of the regulation of IL-6 receptor signaling and T_H_17 development, which was confirmed by shRNA-mediated knockdown and overexpression of each *Traf* gene in differentiating wild-type CD4^+^ T cells. TRAF2 did not inhibit the STAT3 phosphorylation downstream of IL-21 receptor in CD4^+^ T cells (16), confirming the specificity of TRAF2 to the IL-6 receptor signaling. Thus, we concluded that both TRAF2 and TRAF5 work as negative regulators of the IL-6 receptor signaling pathway.

NF-κB-inducing kinase (NIK) is critical for T_H_17 development, and both TRAF2 and TRAF3 limit NIK activity through ubiquitin-dependent degradation (18–21). In this reason, it was possible that TRAF2 and TRAF3 might inhibit T_H_17 development via degradation of NIK. However, increasing or decreasing the expression of TRAF3 did not affect the sensitivity of the IL-6 receptor signaling and the development of T_H_17 cells (16). In addition, it is unclear how TRAF2 regulates the differentiation of naïve CD4^+^ T cells into T_H_17 cells (20). Thus, we concluded that TRAF2 regulation of NIK expression levels is not the mechanism to limit the development of T_H_17 cells.

Although naïve CD4^+^ T cells from *Traf5*^−/−^ and wild-type mice produced equivalent amounts of IL-6 in response to antigen stimulation (15), *Traf2*^−/−^ macrophages and *Traf5*^−/−^ B cells produced more IL-6 in response to TLR stimulation (22, 23). *Traf2*^−/−^ *Tnfa*^−/−^ mice displayed an inflammatory disorder and had elevated levels of IL-6 in serum (20). Thus, TRAF2 and TRAF5 might contribute to the development of T_H_17 cells *in vivo* via negative regulation of IL-6 production.

## Inhibitory role for TRAF2 and TRAF5 in the initial stage of T_H_17 development

**Figure 1:**
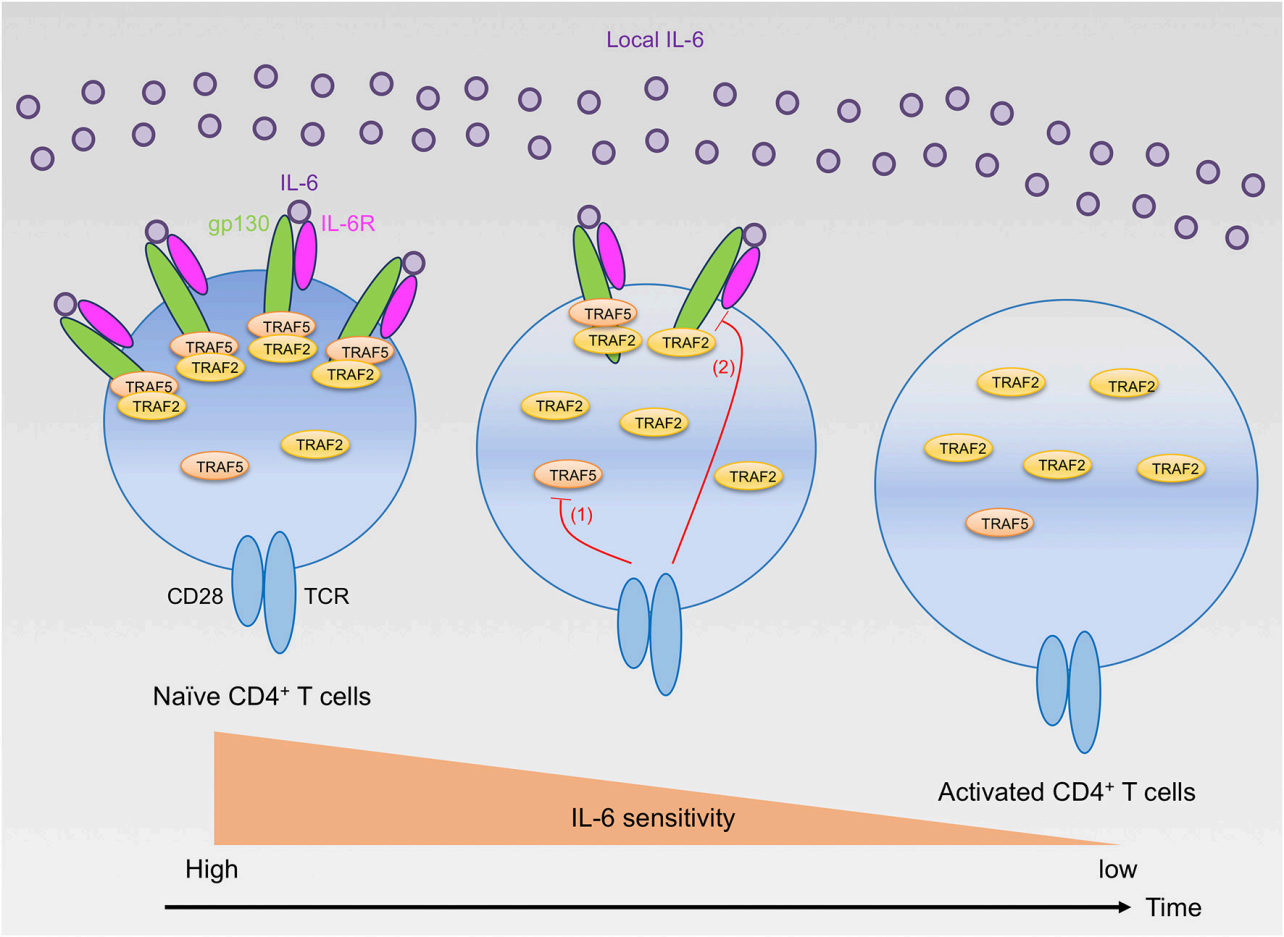
Regulation of IL-6 receptor signaling sensitivity by TRAF2 and TRAF5 in CD4^+^ T cells. Naïve CD4^+^ T cells highly express gp130, IL-6R, TRAF2, and TRAF5. Naïve CD4^+^ T cells can react to extracellular IL-6, but the signaling via the IL-6 receptor, IL-6R and gp130, is restrained by gp130-associated TRAF2 and TRAF5. After T cell activation by TCR and CD28, TRAF5 protein is rapidly downregulated (red line 1), while TRAF2 protein is maintained. Hence, TRAF5 limits the early IL-6 receptor signaling that is important for T_H_17 development. In contrast, TRAF2 can inhibit the signaling activity of the IL-6 receptor complex even in the later phase of T_H_17 differentiation. Moreover, the TCR and CD28 signaling also suppresses the expression of both gp130 and IL-6R (red line 2), and these receptor proteins are almost disappeared from the T cell surface within a few days after T cell activation. Therefore, activated CD4^+^ T cells lose their responsiveness to IL-6 and cannot receive the instructive IL-6 receptor signals required for T_H_17 development.

While TRAF2 and TRAF5 seemed to exhibit a similar role for the IL-6 receptor signaling pathway, detailed analyses revealed that the inhibition kinetic of TRAF2 for the IL-6 receptor signaling was different from that of TRAF5 due to different expression kinetics of respective TRAF proteins in developing CD4^+^ T cells. TRAF5 was higly expressed by unactivated naive CD4^+^ T cells, and *Traf5* mRNA and TRAF5 protein were rapidly disappeared within a few hours upon TCR triggering (16). This means that there is only a narrow window of time for the inhibition for IL-6 receptor signaling by TRAF5 in differentiating CD4^+^ T cells. In contrast to this, *Traf2* mRNA and TRAF2 protein were stably detected during the course of CD4^+^ T cell development, implying that TRAF2 can continuously suppress IL-6 receptor signaling as long as both gp130 and IL-6R are expressed in differentiating CD4^+^ T cells. In comparison with the regulation of *Traf2* and *Traf5* mRNAs, the expression of *Traf1, Traf3, Traf4*, and *Traf6* mRNAs in CD4^+^ T cells were oppositely regulated, and these mRNAs were rapidly upregulated after stimulation with TCR and CD28, demonstrating that the expression of respective TRAF molecules is differentially controlled in recently activated naïve CD4^+^ T cells. In addition to this regulatory mechanism of TRAF molecules, after triggering of TCR and CD28, gp130 and IL-6R expressed by naïve CD4^+^ T cells were downregulated in a time-dependent manner, and these molecules were hardly detected on the surface of activated CD4^+^ T cells at 48 h after activation. In agreement with these results, addition of IL-6–sIL-6R at later time points of T_H_ differentiation could not effectively promote the development of T_H_17 cells, and retrovirus-mediated transduction of short hairpin RNA (shRNA) that targets *Traf5* in differentiating wild-type CD4^+^ T cells could not enhance the production of IL-17. On the other hand, shRNA-mediated knockdown of *Traf2* in differentiating CD4^+^ T cells further promoted the development of T_H_17 cells in both wild-type and *Traf5*^−/−^ conditons (16). Thus, it is reasonable to conclude that the instructive signals from IL-6 receptor for T_H_17 development are restricted both by the negative action of TRAF2 and TRAF5 and by the expression levels of gp130, IL-6R and TRAF5 (Figure 1). The expression of TRAF5 protein can be regulated by the ubiquitin proteasome system (21, 24, 25), although it is not known whether this type of regulation of TRAF5 is ongoing in activated naïve CD4^+^ T cells.

## A molecular mechanism of IL-6 receptor signaling that is regulated by TRAF2 and TRAF5

Naïve *Traf5*^−/−^ CD4^+^ T cells expressed the same level of IL-6R and gp130 as wild-type naïve CD4^+^ T cells did, and the TRAF5 expression did not affect the STAT3 activation downstream of IL-10 receptor or IL-21 receptor in CD4^+^ T cells (15). Thus, we thought that TRAF5 directly regulated a key signaling process in the IL-6 receptor complex. Indeed, endogenously expressed TRAF5 constitutively bound to a cytoplasmic region of gp130 in primary CD4^+^ T cells. Co-immunoprecipitation assay using mutant proteins of TRAF5 and gp130 revealed that TRAF5 required its carboxy-terminal TRAF-C domain but not its amino-terminal RING/zinc-finger domains to interact with gp130 and that the TRAF5-C domain associated with a cytoplasmic region from residue 774 to residue 798 of gp130, gp130 (774-798), ^774^Val-Phe-Ser-Arg-Ser-Glu-Ser-Thr-Gln-Pro-Leu-Leu-Asp-Ser-Glu-Glu-Arg-Pro-Glu-Asp-Leu-Gln-Leu-Val-Asp^798^, which contains recognition elements for the TRAF-C domain (26–28). Similarly, TRAF2 bound to the same region in gp130 via the TRAF2-C domain. This cytoplasmic region of gp130 is highly conserved across various species including human (15, 16).

**Figure 2:**
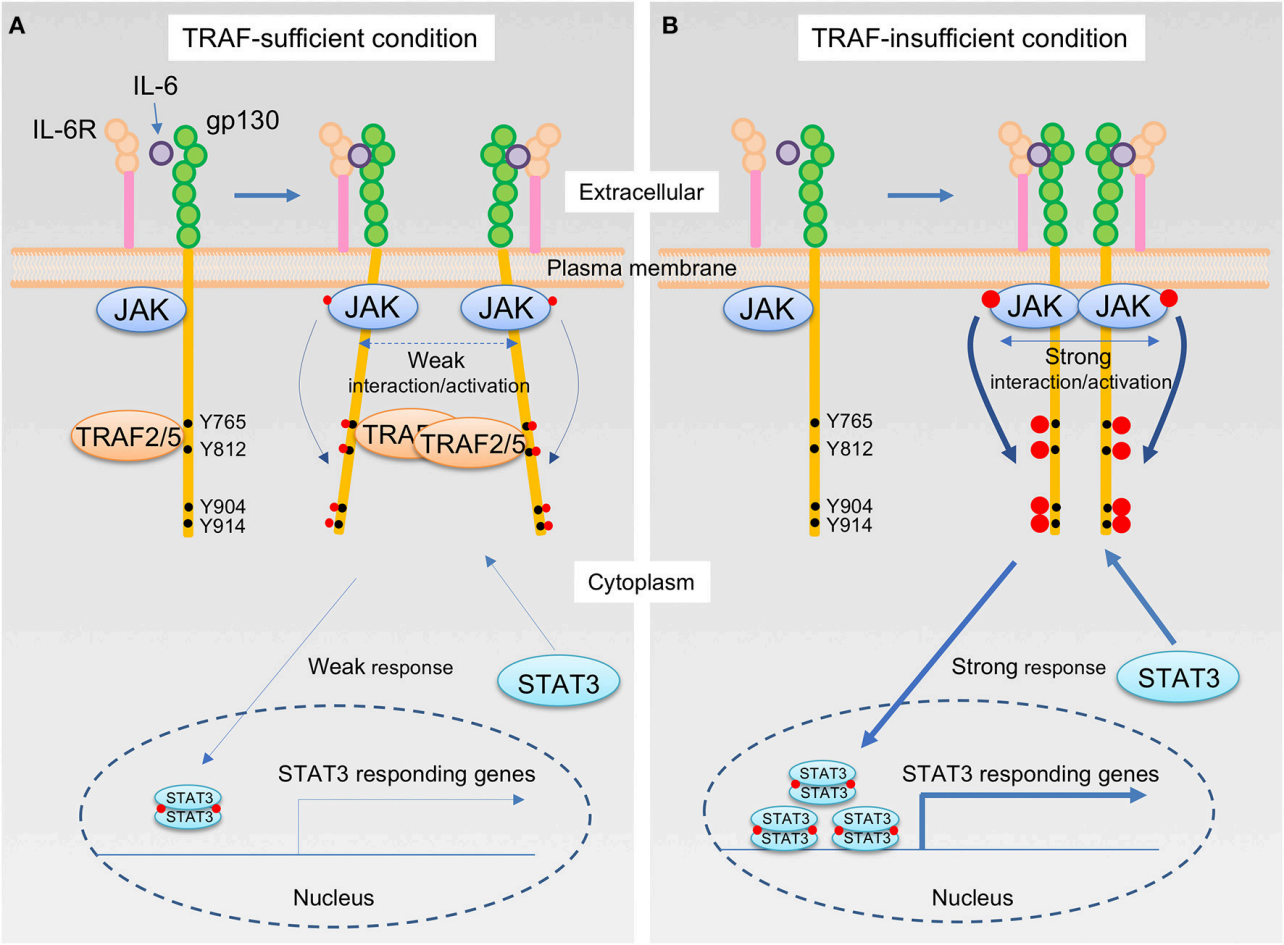
The IL-6 receptor signaling pathway that is regulated by TRAF2 and TRAF5. **(A,B)** Upon interaction of IL-6 with the IL-6R, the complex of IL-6 and IL-6R next binds to the IL-6 receptor common chain gp130, which leads to dimerization of gp130. Janus kinase (JAK) is constitutively bound to the intracellular domains of gp130, and thus this event brings JAKs into close proximity, inducing transphosphorylation of each JAK on a tyrosine residue, indicated in red circle, that stimulates kinase activity of JAKs. The activated JAKs then phosphorylate the cytoplasmic tail of gp130 on specific tyrosine residues, generating binding sites for signal transducer and activator of transcription (STAT) including STAT3. Recruitment of a STAT3 to the phosphorylated gp130 brings the STAT3 close to the activated JAK, which then the activated JAK phosphorylates a tyrosine residue of the STAT3. Phosphorylated STAT3 molecules form a dimer, and STAT3 dimers translocate to the nucleus, then induce the gene transcription involved in T_H_17 differentiation, including RAR-related orphan receptor-γt (RORγt) and IL-17. TRAF2 and TRAF5 constitutively bind to a cytoplasmic region of the gp130, which includes an amino acid sequence ^774^VFSRSESTQPLLDSEERPEDLQLVD^798^ and locates between first two out of four distal phosphorylated tyrosine motifs in gp130, Y765, Y812, Y904, and Y914, that are recognized by STAT3. For this reason, JAK interactions are interrupted by the presence of TRAF2 and/or TRAF5, and this event causes a weaker interaction/activation of JAKs and subsequent attenuated responses in the IL-6 receptor signaling pathway **(A)**. On the other hand, in the absence of TRAF2 and/or TRAF5, a stronger association of JAKs facilitates an augmented JAK activation that leads to the enhanced STAT3 responses in the IL-6 receptor signaling pathway **(B)**.

How do TRAF2 and TRAF5 negatively regulate IL-6 receptor signaling? Although the expression of TRAF5 did not inhibit the interaction between JAK1 and gp130, TRAF5 repressed the phosphorylation of JAK1, gp130, and STAT3 mediated by IL-6–sIL-6R. Therefore, it was hypothesized that TRAF5 limits the proximal interaction of JAK proteins and resulting their auto-phosphorylation by disturbing the optimal dimerization of gp130 upon interaction with IL-6 and IL-6R. By employing luciferase fragment complementation system using fusion proteins of JAK1 with either the N-terminal or the C terminal protein fragment of firefly luciferase, it was revealed that TRAF2 or TRAF5 suppressed JAK1-JAK1 interactions occurring after ligation of gp130 with IL-6–sIL-6R. Importantly, the JAK1-JAK1 interaction was intact in a mutant of gp130 (Δ774-798), which lacks the binding site for TRAF2 and TRAF5, even in the presence of TRAF2 or TRAF5. In addition, it was notable that RING and Zn finger domains of TRAF2 and TRAF5 are dispensable but TRAF-C domain is essential to suppress JAK1-JAK1 interactions to limit IL-6 receptor signaling (15, 17). This demonstrates that TRAF2 and TRAF5 require both TRAF-C domain and TRAF-binding region in gp130 to inhibit JAK1-JAK1 interactions and the following molecular events in the IL-6 receptor signaling pathway. Moreover, the expression of a peptide fragment of gp130 (769–800) fused with GFP in wild-type CD4^+^ T cells promoted the T_H_17 development (15), indicating that the peptide fragment of gp130 competitively inhibits the endogenous interaction between TRAFs and gp130. All of these data support the idea that TRAF2 and TRAF5 associated with gp130 via TRAF-C domains negatively regulate the JAK activation in the IL-6 receptor signaling complex that plays an essential role in initiating the JAK-STAT signaling pathway (Figure 2).

There has been some unsolved issues and controversy about the mechanisms regarding regulation of the IL-6 receptor signaling and the T_H_17 development mediated by TRAF2 and TRAF5. Firstly, when CD4^+^ T cells were stimulated with IL-6–sIL-6R instead of IL-6 alone, TRAF2 and TRAF5 could efficiently inhibit the IL-6 receptor signaling activity (15, 16). IL-6 *trans* signaling is activated via membrane-bound gp130 that interacts with a complex of sIL-6R and IL-6. IL-6 *trans* signaling or IL-6 cluster signaling plays a dominant role for priming pathogenic T_H_17 cells (29, 30). This suggests that TRAF2 and TRAF5 may preferentially restrain IL-6 *trans* signaling activity. Secondly, the identified TRAF-binding region in gp130 is located between first two phosphorylated-tyrosine (p-Tyr) motifs in gp130, and thus it is possible that TRAF2 and TRAF5 may inhibit the binding of STAT3 to these p-Tyr motifs via making steric hindrance in gp130 (15, 16). Thirdly, it is not clear how gp130-associated TRAF proteins inhibit the proximal JAK interaction in the receptor complex. The binding between gp130 and JAK1 occurred independently of the interaction between gp130 and TRAF5 (17). TRAFs may restrain the formation of gp130 dimer and inhibit the reposition process of associated JAKs (Figure 2). Fourthly, TRAF2 and TRAF5 might recruit a protein tyrosine phosphatase to the IL-6 receptor signaling complex to inhibit the JAK-STAT signaling, although this mechanism is utilized by TRAF3 expressed in B cells (31). Fifthly, TRAF5 might work as a positive regulator for RAR-related orphan receptor-γt (RORγt) activity and augment RORγt-mediated T_H_17 responses in a certain experimental setting (32), although this is inconsistent with the conclusion presented here.

## Concluding remarks

It is now clear that TRAF family molecules control a wide range of signaling mediated by membrane-bound receptors in many cell types including CD4^+^ T cells. Findings highlighted here illustrate how TRAF2 and TRAF5 impact IL-6-mediated T_H_17 generation and T_H_17-driven immuno-pathology *in vivo* and *in vitro* and the molecular mechanisms by which TRAF2 and TRAF5 restrain IL-6 receptor signaling. The conclusion that TRAF2 and TRAF5 interacted with gp130 suppress proximal JAK-JAK interactions and resulting JAK phosphorylation in the receptor complex suggests that these TRAFs also regulate signals downstream of receptors for other IL-6 family cytokines that utilize gp130. Dysregulated TRAF5 expression might play an important role in autoimmune and inflammatory diseases in human (33). It will be absolutely important in the future to understand how TRAF2 and TRAF5 control signal transduction through unconventional cytokine receptors and to characterize its impact on immune responses and other relevant biological responses mediated by CD4^+^ T cells and other cell types.

## Author contributions

All authors listed have made a substantial, direct and intellectual contribution to the work, and approved it for publication.

### Conflict of interest statement

The authors declare that the research was conducted in the absence of any commercial or financial relationships that could be construed as a potential conflict of interest.
